# Successful elimination of bilirubin in critically ill patients with acute liver dysfunction using a cytokine adsorber and albumin dialysis: a pilot study

**DOI:** 10.1038/s41598-021-89712-4

**Published:** 2021-05-13

**Authors:** Christina Scharf, Uwe Liebchen, Michael Paal, Andrea Becker-Pennrich, Michael Irlbeck, Michael Zoller, Ines Schroeder

**Affiliations:** 1 Department of Anesthesiology, LMU Hospital, Marchioninistrasse 15, 81377 Munich, Germany; 2 Institute of Laboratory Medicine, LMU Hospital, Munich, Germany

**Keywords:** Liver diseases, Hepatotoxicity

## Abstract

There are different methods of artificial liver support for patients with acute liver dysfunction (ALD). However, CytoSorb (CS) might be a new approved option for those patients. Question of interest is whether the elimination performance of CS was comparable to that of advanced organ support (ADVOS). Patients, treated with CS (integrated into high-flux dialysis) or ADVOS and a total bilirubin > 10 mg/dl were included. Laboratory parameters were evaluated before starting therapy (d0) and 12–24 h thereafter (d1). The Wilcoxon-test with associated samples was used for statistical analysis. Thirty-nine patients (33 CS, 6 ADVOS) were included. The median bilirubin at d0 was 16.9 and 17.7 mg/dl and at d1 was 13.2 and 15.9 mg/dl, in the CS and ADVOS group, respectively. There was a significant bilirubin reduction as well in the CS group (*p* < 0.001, median relative reduction: 22.5%) as in the ADVOS group (*p* = 0.028, median relative reduction: 22.8%). There was no significant difference in the relative bilirubin reduction between CS and ADVOS therapies. The use of CytoSorb and ADVOS in patients with ALD led to a significant and comparable decrease in total bilirubin. The easy use of CS might be an advantage compared to other procedures.

## Introduction

Despite the further development of modern intensive care medicine, acute liver dysfunction (ALD) is associated with high mortality in intensive care unit (ICU) patients^1^. The different entities of liver failure are divided into primary and secondary reasons. Reasons for primary liver dysfunction are hepatotoxic medication or viral infection, leading to an acute liver failure (ALF)^2,3^. More often, liver failure occurs secondarily due to cholestasis and hypoxic liver injury as the most important reasons for ALD in the critically ill^4^. Secondary ALD can also be caused by sepsis or cardiac shock with an acute and possible irreversible dysfunction of the hepatocytes^5^. However, there is no uniform definition of ALD in critically ill patients. In conclusion, both forms can result in coagulopathy, encephalopathy, and increase in cholestasis and liver integration parameters.

The main problem associated with ALD, especially when caused by cholestasis, is that toxic substances, such as bile acids (BAs), are no longer removed from the liver. The accumulation in the blood and liver can result in permanent damage of the hepatocytes^6^. Furthermore, the accumulation of ammonia can lead to cerebral edema with the risk of persistent cerebral injury^7^.

Bilirubin is a surrogate parameter for cholestatic impairment and may reflect impaired biliary secretion^8^. Horvatits et al. showed that the level of BAs is correlated with bilirubin serum levels in the critically ill. Thus, bilirubin can be used to estimate the amount of BA, because its quantification is less widely established than the quantification of bilirubin^9^. Hence, critically ill patients, with higher bilirubin levels in the blood, were found to have higher mortality compared to those with normal bilirubin levels^10,11^.

The main aim in the treatment of ALD is to prevent patients from permanent hepatocyte damage, which often leads to liver transplantation or death^12^. Therefore, metabolic disorder, coagulation disorder, neurological disorder, and sepsis caused by different pathogens should be treated as effectively as possible as a supportive therapeutic concept^13^.

In addition to these regimens, liver support systems are an option for supportive therapy. The concept of these systems is to clear blood of hepatotoxic substances, such as cytokines, BA, and endotoxins to avoid further damage of the hepatocytes. A recently published systematic meta-analysis showed evidence of the reduction in mortality with liver support systems^14^. However, different systems are available. One opportunity is to implement plasma exchange, which can improve outcomes^15^, but is expensive and can lead to hypotension and bleeding complications^16,17^. Another approach is albumin dialysis, for example, with Advanced Organ Support (ADVOS) or MARS^18,19^. ADVOS is an advanced hemodialysis system combining different forms of organ support (kidney, lung, liver) using three circuits: the extracorporeal blood circuit, the dialysate circuit, and the albumin multi circuit. It aims to remove water-bound and albumin-bound toxins, in addition to CO_2_, and correct acid–base disturbances. Although case studies have shown this effect, it has not yet been established in daily intensive care practice^20,21^.

Since 2017, case reports have described the elevation of bilirubin and BA and the support of liver excretory function using the cytokine adsorber CytoSorb (CS)^22–24^. Furthermore, Calabró et al*.* published the first clinical trial that evaluated the effect of CS on bilirubin levels in 40 critically ill patients^25^. In 2019, Gemelli et al*.* described the in vitro kinetics of the elimination of bilirubin via CS as a novel tool that can be used in patients with ALD^26^.

In the context of clinical routine, ADVOS dialysis and CS were used at two ICUs at the LMU hospital in patients with ALD. There is a lack of data assessing the effect of CS in patients with ALD, particularly compared with other liver support systems such as ADVOS. Therefore, a retrospective data analysis was performed including patients between April 2015 and May 2020. The effectiveness of both systems, particularly with respect to total bilirubin elimination, was evaluated and compared.

## Methods

### Study setting

This was a monocentric, retrospective observational study investigating the influence of ADVOS and CS therapy in critically ill patients with acute liver dysfunction (ALD). Patients were included between April 2015 and May 2020 during their stay at two ICUs at the LMU hospital, Munich.

### Laboratory testing and data collection

All clinical chemical parameters were determined with standard clinical chemistry tests at the institute of laboratory medicine. For data evaluation, demographic data, clinical variables, and laboratory variables were collected from the laboratory and patient information system. Baseline characteristics (age, gender, body mass index (BMI), extracorporeal membrane oxygenation (ECMO), 7-day mortality, in-hospital mortality, sequential organ failure assessment (SOFA) score, Simplified Acute Physiology Score (SAPS), model end stage liver disease (MELD) score, norepinephrine demand, reason for admission to ICU, and reason for ALD were evaluated on the treatment day. Laboratory data were collected one day before and during the intervention.

### Study population

**Figure 1:**
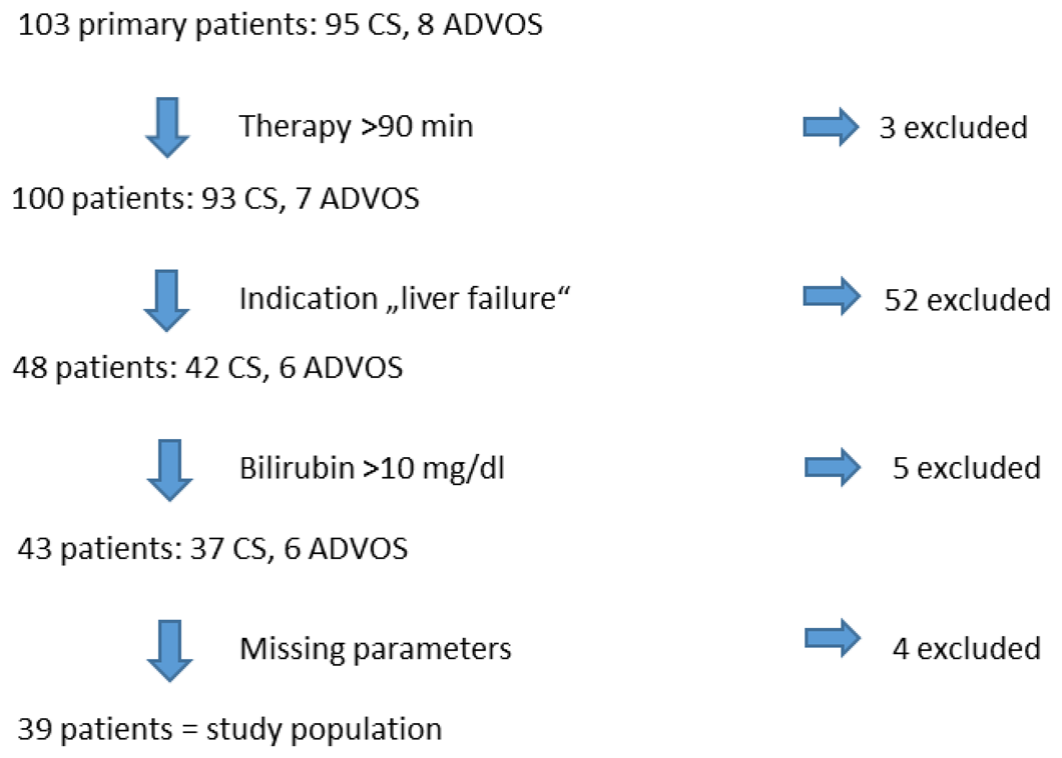
Selection of the study population. CS: CytoSorb, ADVOS: advanced organ support.

All patients, who were treated with CS (integrated into high-flux dialysis (Fresenius Ultraflux AV 600S, surface area 1.4 m^2^)) or ADVOS, were screened for evaluation. Patients were only included in the study population when the therapy was longer than 90 min, total bilirubin level in the serum was > 10 mg/dl before the start of therapy, and bilirubin was measured 12–24 h after starting therapy. Figure 1 shows, based on the screened primary patients, the patients excluded in each case by the exclusion criteria. The remaining patients are the study population presented below. Regardless of the number of treatments with CS or ADVOS received by patients during their stay at the ICU, only the first treatment with the indication “acute liver dysfunction or hyperbilirubinemia” was evaluated. The treating physician was responsible for establishing the indication and selecting the liver replacement procedure.

### Blood sampling

In the data evaluation, three timepoints were considered depending on CS or ADVOS treatment:

- d−1: 24–36 h before start of ADVOS/CS
- d0: 0–12 h before the start of ADVOS/CS
- d1: 12–24 h after starting ADVOS/CS (= end of ADVOS/CS)

### Statistical analysis

Statistical analysis was performed with IBM SPSS statistics (Version 26.0. IBM Corp., Armonk, NY, USA) and Python (Version 3.5.2, Packages Pandas 0.24.2 and scipy 1.4.1). Because the laboratory variables did not have a normal distribution according to the Shapiro–Wilk test, non-parametric tests were used. Continuous variables are given as median and interquartile range (IQR) or with minimum and maximum. *U*-test was used to identify differences in baseline parameters in both groups. The effect of CS and ADVOS treatment on the reduction in total bilirubin, serum alanine aminotransferase (ALT), serum aspartate aminotransferase (AST), glutamine-glutamyl transferase (GGT), alkaline phosphatase (AP), norepinephrine demand, and SAPS II was investigated. To describe the change of these parameters before and during blood purification, the Wilcoxon test with associated samples was used. Furthermore, it was used to identify differences in the bilirubin elimination of CS and ADVOS patients. The relative change of the parameters (%) was calculated with: 100 − ((100/parameter d0) * parameter d1).

### Ethics approval and consent to participate

Ethical approval was obtained from the ethical review committee of the Ludwig-Maximilians-Universität (registration number 20-477). Data collection was in accordance with the review board and therefore with all valid guidelines. Informed consent of the patients is given as part of the ethics approval process.

## Results

### Demographic and clinical data

In total, 39 patients (33 CS, 6 ADVOS) were included in the evaluation. Reason for admission to the ICU in patients treated with CS was, in descending order: ARDS (24.2%), septic shock (15.2%), polytrauma (15.2%), liver transplantation (12.1%), ALF (12.1%), lung transplantation (6.1%), cardiogenic shock (6.1%), and other reasons (9.0%). The reasons for admission in patients treated with ADVOS were acute liver failure (50.0%), cardiogenic shock (33.3%), and liver transplantation (16.7%). The reasons for ALD in both groups were, in descending order: septic multiorgan failure (25.6%), hypoxic liver failure (23.1%), acute-on-chronic liver failure (17.9%), liver graft failure (12.8%), secondary sclerosing cholangitis (10.3%), and different reasons (10.3%). Seven patients had a preexisting liver cirrhosis. The reasons for hypoxic liver failure were hemorrhagic shock, cardiogenic shock and pulmonary embolism. The median age was 55 years and 69% of the patients were male. An ECMO therapy was necessary in 29% of the patients and the in—hospital mortality was 82%. The median SAPS II at the treatment day (d0) was 80 points, indicating a mortality of 92.5%. The median MELD score before therapy was 35 points. Detailed patient characteristics are displayed in Table 1.

**Table 1:** Patient characteristics.

	All patients: n (%) or median [range: min, max]	CytoSorb: n (%) or median [Range: min, max]	ADVOS: n (%) or median [Range: min, max]	Differences in both groups (*p*-value)
No. of patients	39 (100)	33 (100)	6 (100)	
Age, years	55 [18, 80]	55 [18, 76]	46 [19, 80]	0.84
Gender, male/female	27 (69.2) / 12 (30.8)	23 (69.7)/10 (30.3)	4 (66.7) / 2 (33.3)	0.92
BMI, kg/m^2^	27.7 [16.2, 55.1]	27.8 [16.2, 55.1]	26.0 [18.8, 35.2]	0.56
ECMO therapy	11 (28.2)	10 (30.3)	1 (16.7)	0.61
7-days mortality	12 (30.8)	10 (30.3)	2 (33.3)	0.92
In-hospital mortality	32 (82.1)	28 (84.8)	4 (66.7)	0.51
SOFA score d0	20 [12, 23]	20 [12, 23]	21 [18, 23]	0.48
SAPS II d0	80 [47, 111]	80 [47, 109]	72 [50,111]	0.53
MELD score d0	35.0 [25.4, 52.9]	33.6 [25.4, 52.9]	38.5 [30.5, 44.8]	0.10
MELD score d1	34.4 [25.8, 52.3]	33.8 [25.8, 52.3]	38.5 [30.1, 45.8]	0.23
Total bilirubin (mg/dl) d-1	16.8 [4.2, 40.2]	14.2 [4.2, 40.2]	18.5 [11.7, 26.9]	0.35
Total bilirubin (mg/dl) d0	17.3 [10.2, 41.1]	16.9 [10.2, 41.1]	17.7 [10.7, 28.0]	0.89
Total bilirubin (mg/dl) d1	13.3 [5.2, 25.6]	13.2 [5.2, 25.6]	15.9 [6.8, 21.9]	0.81

SOFA, sequential organ failure assessment; SAPS, Simplified Acute Physiology Score; ADVOS, advanced organ support; BMI, body mass index; ECMO, extracorporeal membrane oxygenation.

### Effect of CytoSorb and ADVOS on bilirubin elimination

**Figure 2:**
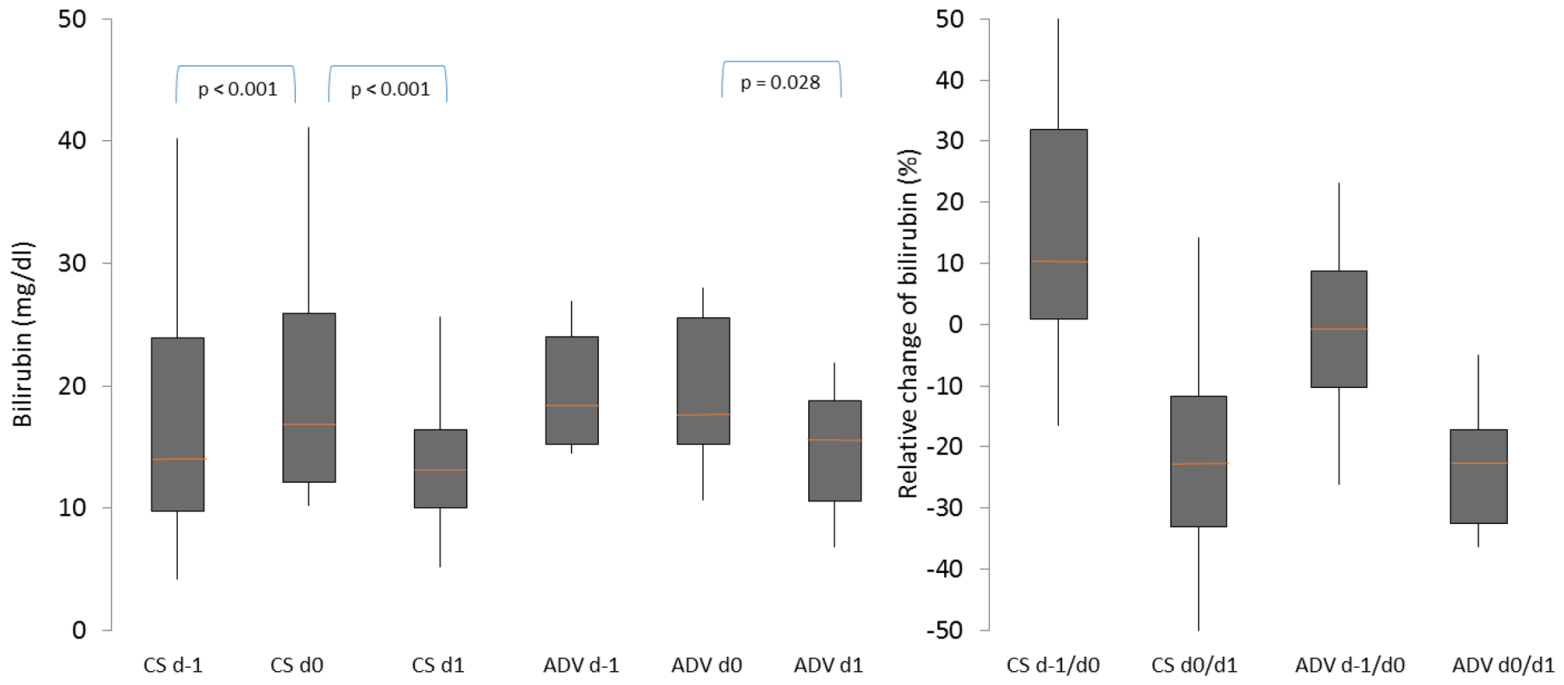
Development and relative reduction in bilirubin levels in patients with CytoSorb and ADVOS therapy. d–1: day before treatment, d0: shortly before treatment, d1: directly after treatment, CS: CytoSorb, ADV: advanced organ support; orange line represents the median, grey boxes the interquartile range and the whiskers are limited to 1.5 times the interquartile range.

The relative change (%) in total bilirubin during the time periods (d − 1/d0) and (d0/d1) for both CS and ADVOS therapy was analyzed. There was a significant increase in total bilirubin in the period prior to CS treatment (*p* < 0.001) with a median relative increase of 10.3% (IQR: 8.8, 31.9%). In contrast, there was no significant change of total bilirubin in the period prior to ADVOS therapy (*p* = 0.917) with a median relative change of − 6.6% (IQR: − 10.2, 8.8%). There was also a significant decrease in total bilirubin during CS therapy (*p* < 0.001; median relative change 22.5% (IQR: 11.8, 33.1%)) and during ADVOS treatment (*p* = 0.028; median relative change 22.8% (IQR: 17.1, 32.5%)). However, there was no significant difference in bilirubin elimination in both groups. Figure 2 shows the course of total bilirubin and relative change of bilirubin prior and during CS and ADVOS therapies as box plots.

### Changes of other parameters during blood purification

Other relevant parameters in patients with ALD are ALT, AST, GGT, and AP. Moreover, relevant outcome parameters are the reduction of norepinephrine, and the reduction of SAPS II. The change in these parameters during CS therapy and ADVOS therapy (d0–d1) was analyzed. Table 2 displays both the primary data and the statistical results. There was a significant reduction in ALT (*p* = 0.007), AST (*p* = 0.016), GGT (*p* = 0.013), SAPS II (*p* = 0.01) and norepinephrine demand (*p* = 0.012) in patients treated with CS. A significant change was not seen in patients with ADVOS therapy.

**Table 2:** Changes in different parameters during blood purification.

Parameter	CS therapy				ADVOS therapy			
	Mean	SD	z-value	*p*-value	Mean	SD	z-value	*p*-value
ALT (U/l) d0	614	1707			620	1067		
ALT (U/l) d1	395	1112			462	725		
ΔALT (d0–d1)	219	715	− 2.68	0.007*	159	344	− 0.52	0.600
AST (U/l) d0	1512	4338			1211	2525		
AST (U/l) d1	1033	3003			951	1827		
ΔAST (d0–d1)	479	1447	− 2.41	0.016*	260	709	− 0.31	0.753
GGT (U/l) d0	307	392			369	398		
GGT (U/l) d1	276	375			356	392		
ΔGGT (d0–d1)	32	78	− 2.50	0.013*	13	37	− 0.67	0.500
AP (U/l) d0	390	92						
AP (U/l) d1	364	81						
ΔAP (d0–d1)	26	67	− 1.19	0.233				
SAPS II d0	79	14			76	20		
SAPS II d1	73	11			77	17		
ΔSAPS II (d0–d1)	6	9	− 3.3	0.01*	− 1	6	− 0.3	0.79
Nor (mg/dl) d0	2.0	2.5			2.0	1.5		
Nor (mg/dl) d1	1.4	1.2			1.5	1.4		
ΔNor (d0–d1)	0.6	1.7	− 2.5	0.012*	0.5	1.3	− 0.81	0.41

CS: CytoSorb, ADVOS: advanced organ support, CI: confidence interval, SD: standard deviation, ALT: serum alanine aminotransferase, AST: serum aspartate aminotransferase, GGT: glutamine-glutamyl transferase, AP: alkaline phosphatase, SAPS: Simplified Acute Physiology Score, Nor: norepinephrine demand, d: day.

## Discussion

ALD is a common syndrome in critically ill patients with a variety of underlying conditions^8^. The increase in total bilirubin can be an expression of cholestasis or acute damage of the hepatocytes, and the sources causing the dysfunction are often not directly treatable. Therefore, symptomatic therapy is of great importance^13^. Since there is no clear description of ALD in the literature and liver replacement procedures are only considered in cases of severe hepatic impairment, we used a severely elevated bilirubin of  > 10 mg/dl as an inclusion criterion. Due to this inclusion criterion, only extremely ill patients were included in our study population. This is reflected in the high median MELD score of 35 points, the high median SAPS II of 80 points, and an in-hospital mortality rate of 82%. Therefore, the mortality rate was lower than expected according to SAPS II (expected mortality: 92.5%). However, in the absence of a control group, it is not possible to make a statement about a reduction in mortality with the use of liver support systems. Nevertheless, other authors also dare to compare actual mortality with predicted mortality and also see lower values in observed mortality, as our study did^27^.

In contrast to newborns, hyperbilirubinemia in adult patients with an intact blood–brain barrier does not lead to direct toxic side effects^28^. However, bilirubin is an easy to measure surrogate parameter for the accumulation of BA in the critically ill^29^, because BA cannot be routinely determined, even in large clinics, due to a less widely established method^30^. An increase in BA can lead to potentially permanent damage to the hepatocytes, particularly in the case of cholestasis, endothelial injury in the kidney and lung tissues, and destruction of the erythrocytes^31,32^. Therefore, the use of liver support systems is very appropriate in cases of severe hepatic dysfunction.

Various blood purification systems are available as supportive therapeutic measures to eliminate toxic substances in patients with ALD^33^. In particular, methods of albumin dialysis have been frequently used in the past^19^. A recent publication on the use of ADVOS describes a moderate but significant decrease in bilirubin (6.9 → 6.5 mg/dl). This could also be due to the fact that bilirubin was only modestly elevated at baseline^27^. However, these systems are complex and expensive. In addition a survival benefit has not been demonstrated in a randomized study population^34,35^. This might explain why few patients have been treated with ADVOS at our ICU. However, a recently published meta-analysis on different liver support systems showed a reduction in mortality and an improvement in hepatic encephalopathy without favoring a special kind of device^14^. Therefore, it is important to know whether a new therapeutic option such as CS works at least as well as proven approaches such as ADVOS. According to the manufacturer's information, CS has a surface area of about 45,000 m^2^ and can eliminate molecules up to a size of 55 kDa. This includes the substances ammonia, bilirubin, and most bile acids^26,33^. However, with a molecule size of only 17 Da, ammonia can easily be removed by high-flux dialysis^36^. Since ammonia was not determined frequently enough in routine clinical practice, no statement can be made on this in our study. In contrast, although a significant reduction in AST (92 kDa), ALT (110 kDa), and GGT (64 kDa) occurred during CS treatment, these substances cannot be adsorbed by CS due to their molecular size. Therefore, the significant decrease during CS treatment might already reflect an improvement of liver function. In-vivo studies with measurement of these substances at the adsorbers’ in- and outlet could be useful to rule out clearance by CS. An elimination of transaminases also cannot be assumed to occur with ADVOS therapy. The absence of a reduction during ADVOS therapy might be explained by the small sample size.

In addition to the lower than expected mortality and the reduction in bilirubin, there was also a significant reduction in norepinephrine demand with the use of CS, resulting in hemodynamic stabilization. This could subsequently lead to better perfusion of the liver and thus also, if given, control the cause of ALD. The significant reduction in SAPS II during CS is another indicator of an improvement in the patient's outcome.

Our study showed that total bilirubin was effectively removed in both groups. Because there was no significant difference between the procedures, they can be considered to be at least equivalent in this respect. There was a significant increase in bilirubin prior to CS therapy, whereas bilirubin decreased in the ADVOS group. An increase in bilirubin before therapy could be indicative of worsening liver dysfunction. Therefore, it is possible that we have underestimated the effect of CS on the reduction in bilirubin because further accumulation during CS might occur due to ongoing hepatic injury. However, the data available on the therapy of ALD in general, and the use of blood purification systems in particular, remain insufficient^17,20,37^. Precisely because alternative systems are complex and difficult to use, CS could be a user-friendly alternative that can be easily integrated in, for example, high-flux dialysis. Recently, Tomescu et al. showed an improvement in liver functional tests using CS in patients with ALF^38^. Since 2019, CS has had a CE-mark (marking of conformity for medical devices) for the elimination of bilirubin^22,33^.

The present study has several limitations. Liver support therapy and measurement of laboratory parameters was performed in clinical routines, so a slight deviation of timepoints cannot be excluded. Furthermore, total bilirubin was used as a surrogate parameter for the BA level, because quantification was not available. A prospective study describing the effect of BA elimination would be desirable. Moreover, small amounts of total bilirubin can also be eliminated by high-flux dialysis and the elimination was therefore a combination of CS therapy and high-flux dialysis. Because there was no control group, advantages in terms of faster recovery of liver function or mortality cannot be established, and spontaneous changes in liver function cannot be assessed. It should also be noted that due to the difference in size of the groups and the lack of randomization, a significant difference between the two groups might not have been detected. Additionally, ADVOS was only available until 2017, so the treating physician had no longer the opportunity to choose between both systems. Furthermore, as data collection was over five years, a change in treatment decisions over time is possible. However, as guidelines did not differ in terms of liver support systems over the study period, different patient selection can be ruled out.

## Conclusion

Liver support systems play an important role in the supportive therapy of patients with ALD, although they are often difficult to use and have many side effects. The use of ADVOS and CytoSorb (integrated into high-flux dialysis) led to a significant and comparable decrease in bilirubin in critically ill patients. An advantage of CS is its easy integration into high-flux dialysis, which allows its use at smaller hospitals. Prospective studies should follow to demonstrate further benefits of liver support, such as a decrease in mortality in patients with ALD.

## Data Availability

All data generated during this study are included in this article.

## Abbreviations

ADVOS: Advanced organ support
ALD: Acute liver dysfunction
ALF: Acute liver failure
ALT: Serum alanine aminotransferase
AP: Alkaline phosphatase
AST: Serum aspartate aminotransferase
BA: Bile acid
BMI: Body mass index
ECMO: Extracorporeal membrane oxygenation
GGT: Glutamine-glutamyl transferase
ICU: Intensive care unit
MELD: Model end stage liver disease
SAPS: Simplified acute physiology score
SOFA: Sequential organ failure assessment

## References

[1] Reuben A (2016). Outcomes in adults with acute liver failure between 1998 and 2013: an observational cohort study. Ann. Intern. Med..

[2] Stravitz RT, Lee WM (2019). Acute liver failure. Lancet.

[3] Real M (2019). Drug-induced liver injury: highlights of the recent literature. Drug Saf..

[4] Horvatits T, Trauner M, Fuhrmann V (2013). Hypoxic liver injury and cholestasis in critically ill patients. Curr. Opin. Crit. Care.

[5] Lelubre C, Vincent JL (2018). Mechanisms and treatment of organ failure in sepsis. Nat. Rev. Nephrol..

[6] Li T, Apte U (2015). Bile acid metabolism and signaling in cholestasis, inflammation, and cancer. Adv. Pharmacol..

[7] Kerbert AJ, Engelmann C, Jalan R (2018). Neurocritical care management of hepatic encephalopathy and coma in liver failure. Semin. Respir. Crit. Care Med..

[8] Jenniskens M (2016). Cholestatic liver (dys)function during sepsis and other critical illnesses. Intensive Care Med..

[9] Horvatits T (2019). Liver injury and failure in critical illness. Hepatology.

[10] Vitek L (2019). Association between plasma bilirubin and mortality. Ann. Hepatol..

[11] Kramer L (2007). Incidence and prognosis of early hepatic dysfunction in critically ill patients—a prospective multicenter study. Crit. Care Med..

[12] Olivo R, Guarrera JV, Pyrsopoulos NT (2018). Liver transplantation for acute liver failure. Clin. Liver Dis..

[13] Khan R, Koppe S (2018). Modern management of acute liver failure. Gastroenterol. Clin. North Am..

[14] Alshamsi F (2020). Extracorporeal liver support in patients with liver failure: a systematic review and meta-analysis of randomized trials. Intensive Care Med..

[15] Larsen FS (2016). High-volume plasma exchange in patients with acute liver failure: an open randomised controlled trial. J. Hepatol..

[16] Yuan S (2018). Therapeutic plasma exchange: a prospective randomized trial to evaluate 2 strategies in patients with liver failure. Transfus. Apher. Sci..

[17] Stahl K (2019). Therapeutic plasma exchange in acute liver failure. J. Clin. Apher.

[18] Tsipotis E, Shuja A, Jaber BL (2015). Albumin Dialysis for liver failure: a systematic review. Adv. Chronic Kidney Dis..

[19] Falkensteiner C (2020). Comparison of the albumin dialysis devices molecular adsorbent recirculating system and ADVanced organ support in critically ill patients with liver failure—a retrospective analysis. Ther. Apher Dial.

[20] Fuhrmann V (2020). Advanced organ support (ADVOS) in the critically ill: first clinical experience in patients with multiple organ failure. Ann. Intensive Care.

[21] Huber W (2017). First clinical experience in 14 patients treated with ADVOS: a study on feasibility, safety and efficacy of a new type of albumin dialysis. BMC Gastroenterol..

[22] Acar U (2019). Impact of cytokine adsorption treatment in liver failure. Transpl. Proc..

[23] Dhokia VD (2019). Novel use of Cytosorb haemadsorption to provide biochemical control in liver impairment. J. Intensive Care Soc..

[24] Piwowarczyk P (2019). Hemoadsorption in isolated conjugated hyperbilirubinemia after extracorporeal membrane oxygenation support. Cholestasis of sepsis: a case report and review of the literature on differential causes of jaundice in ICU patient. Int. J. Artif. Organs.

[25] Calabro MG (2019). Blood purification with CytoSorb in critically Ill patients: single-center preliminary experience. Artif. Organs.

[26] Gemelli C (2019). Removal of bilirubin with a new adsorbent system: in vitro kinetics. Blood Purif..

[27] Fuhrmann V (2021). Registry on extracorporeal multiple organ support with the advanced organ support (ADVOS) system: 2-year interim analysis. Medicine (Baltimore).

[28] Watchko JF (2016). Bilirubin-induced neurotoxicity in the preterm neonate. Clin. Perinatol..

[29] Horvatits T (2017). Circulating bile acids predict outcome in critically ill patients. Ann. Intensive Care.

[30] Amplatz B (2017). Bile acid preparation and comprehensive analysis by high performance liquid chromatography-high-resolution mass spectrometry. Clin. Chim. Acta.

[31] Lang E (2016). Bile acid-induced suicidal erythrocyte death. Cell Physiol. Biochem..

[32] Perez MJ, Briz O (2009). Bile-acid-induced cell injury and protection. World J. Gastroenterol..

[33] Dominik A, Stange J (2020). Similarities, differences, and potential synergies in the mechanism of action of albumin dialysis using the MARS albumin dialysis device and the CytoSorb hemoperfusion device in the treatment of liver failure. Blood Purif..

[34] Niewinski G (2020). Intermittent high-flux albumin dialysis with continuous venovenous hemodialysis for acute-on-chronic liver failure and acute kidney injury. Artif. Organs.

[35] Gerth, H.U., et al., *Molecular adsorbent recirculating system (MARS) in acute liver injury and graft dysfunction: Results from a case-control study.* PLoS One, 2017. **12**(4): p. e0175529. 10.1371/journal.pone.0175529 PMC5389829 28403210

[36] Cardoso FS (2018). Continuous renal replacement therapy is associated with reduced serum ammonia levels and mortality in acute liver failure. Hepatology.

[37] Chiu A, Fan ST (2006). MARS in the treatment of liver failure: controversies and evidence. Int. J. Artif. Organs.

[38] Tomescu D *et**al.* Haemoadsorption by CytoSorb(R) in patients with acute liver failure: a case series*.**Int**J**Artif**Organs*, 2020, p. 391398820981383. 10.1177/0391398820981383 33302765

